# Genetic Liability to Rheumatoid Arthritis in Relation to Coronary Artery Disease and Stroke Risk

**DOI:** 10.1002/art.42239

**Published:** 2022-08-17

**Authors:** Shuai Yuan, Paul Carter, Amy M. Mason, Fangkun Yang, Stephen Burgess, Susanna C. Larsson

**Affiliations:** ^1^ Karolinska Institutet Stockholm Sweden; ^2^ University of Cambridge Cambridge UK; ^3^ Ningbo First Hospital and Zhejiang University Ningbo China; ^4^ Karolinska Institutet, Stockholm, Sweden, and Uppsala University Uppsala Sweden

## Abstract

**Objective:**

To assess the causality of the associations of rheumatoid arthritis (RA) with coronary artery disease (CAD) and stroke using the Mendelian randomization approach.

**Methods:**

Independent single‐nucleotide polymorphisms strongly associated with RA (n = 70) were selected as instrumental variables from a genome‐wide association meta‐analysis including 14,361 RA patients and 43,923 controls of European ancestry. Summary‐level data for CAD, all stroke, any ischemic stroke and its subtypes, intracerebral hemorrhage (ICH), and subarachnoid hemorrhage were obtained from meta‐analyses of genetic studies, international genetic consortia, the UK Biobank, and the FinnGen consortium. We obtained summary‐level data for common cardiovascular risk factors and related inflammatory biomarkers to assess possible mechanisms.

**Results:**

Genetic liability to RA was associated with an increased risk of CAD and ICH. For a 1‐unit increase in log odds of RA, the combined odds ratios were 1.02 (95% confidence interval [1.01, 1.03]; *P* = 0.003) for CAD and 1.05 (95% confidence interval [1.02, 1.08]; *P* = 0.001) for ICH. Genetic liability to RA was associated with increased levels of tumor necrosis factor and C‐reactive protein (CRP). The association with CAD was attenuated after adjustment for genetically predicted CRP levels. There were no associations of genetic liability to RA with the other studied outcomes.

**Conclusion:**

This study found that genetic liability to RA was associated with an increased risk of CAD and ICH and that the association with CAD might be mediated by CRP. The heightened cardiovascular risk should be actively monitored and managed in RA patients, and this may include dampening systemic inflammation.

## INTRODUCTION

Rheumatoid arthritis (RA) is the most common autoimmune arthritis, with a prevalence of 1%, and cardiovascular disease (CVD) is the leading cause of mortality worldwide (1, 2). Interestingly, CVD risk is substantially increased in RA and to a similar extent as other established risk factors such as diabetes mellitus (3). In meta‐analyses, both cardiovascular morbidity and mortality have been found to be 1.5‐fold elevated in RA compared to the general population (4, 5). The reasons for this remain poorly understood but may relate to shared risk factors (e.g., obesity and smoking) or an influence of RA on traditional cardiovascular risk factors (e.g., side effects of antirheumatic therapies or reduced physical activity due to pain). Importantly though, traditional risk factors do not fully explain the augmented CVD risk in RA, and observational studies suggest that RA may be a novel and independent risk factor for coronary disease (6, 7, 8, 9). CVD and RA have overlapping pathophysiologic mechanisms which may contribute, such as systemic inflammation, with cytokines raised in RA known to be important in driving atherosclerotic diseases (10). Consistent with this, systemic markers of inflammation are associated with cardiovascular risk in RA (11, 12). However, previous observational studies may have been limited by residual confounding or reverse causality. As such, whether RA is an independent and causal risk factor for CVDs and cardiometabolic risk factors remains equivocal.

Mendelian randomization (MR) analysis is an epidemiologic approach that can strengthen causal inference by using genetic variants as instrumental variables for the exposure (13). The method can minimize the influence of residual confounding, since genetic variants are randomly distributed at conception and are therefore unrelated to self‐adopted lifestyle and environmental confounders (13). In addition, the method can diminish reverse causality because the germline genotype cannot be modified by the onset and progression of the disease (13). Here, we conducted a 2‐sample MR study to examine the associations of genetic predisposition to RA with coronary artery disease (CAD), stroke, and its subtypes and cardiometabolic risk factors. We aimed to provide important evidence regarding the causal role of RA in causing a range of CVD and whether this could be through influencing traditional risk factors or systemic inflammation.

## MATERIALS AND METHODS

### Study design

We first examined the genetic correlations and MR associations of genetic predisposition to RA with CAD and stroke and its subtypes. To assess potential mechanisms, we investigated the associations of genetic predisposition to RA with common cardiovascular risk factors and related inflammatory biomarkers. We then conducted multivariable MR analysis to examine the mediation effects of RA‐associated factors in the associations between genetic predisposition to RA and the cardiovascular end points. This study was based on summary‐level data from international consortia, the UK Biobank, and the FinnGen consortium. All included studies had obtained ethical permits from corresponding ethics committees. The UK Biobank received ethical permits from the North West Multi‐centre Research Ethics Committee, the National Information Governance Board for Health and Social Care in England and Wales, and the Community Health Index Advisory Group in Scotland. All participants provided written informed consent. The present MR analyses were approved by the Swedish Ethical Review Authority (no. 2019‐02793). This study was conducted in accordance with the MR guideline (14).

### Instrumental variable selection

Single‐nucleotide polymorphisms (SNPs) strongly associated with RA (*P* < 5 × 10^−8^) were obtained from a genome‐wide association meta‐analysis that included 14,361 RA patients and 43,923 controls of European ancestry (15). All RA cases were defined by the 1987 criteria of the American College of Rheumatology for RA diagnosis (16) or by a rheumatologist (15). Linkage disequilibrium in selected SNPs was estimated using the 1000 Genomes European reference panel. SNPs in high linkage disequilibrium (*r* ^2^ > 0.01 or clump windows <10,000 kb) were excluded, and the SNP with the lowest *P* value for the genome‐wide association with RA was retained. A total of 70 independent SNPs with beta and SE coefficients scaled to log‐transformed odds of RA were used as instrumental variables (Supplementary Table 1, on the *Arthritis & Rheumatology* website at https://onlinelibrary.wiley.com/doi/10.1002/art.42239). To provide estimates with a more intuitive interpretation, we estimated absolute genetic associations with RA using linear regression and used these summary‐level data for SNP–RA associations in a supplementary analysis (Supplementary Table 1). This enables the calculation of MR estimates that represent odds ratios (ORs) for the studied CVDs per 1% increase in the absolute probability of RA. Genetic associations were estimated in participants of genetic European descent in the UK Biobank. The outcome was defined using electronic health records and International Classification of Diseases codes (ICD‐9 714.0, ICD‐10: M05 or M06). Linear regression was performed with adjustment for age, sex, and 10 genomic principal components.

### Data sources for outcomes

Summary‐level data for the associations of RA‐associated SNPs with CAD, all stroke, any ischemic stroke and its subtypes, ICH, and subarachnoid hemorrhage were obtained from meta‐analyses of genetic studies, international genetic consortia (17, 18, 19, 20), the UK Biobank, and the FinnGen consortium (21). There was minimal sample overlap between the exposure and outcome data sets. Detailed information, including case and control number and covariates adjusted for in the genome‐wide association analysis, is shown in Table 1. The associations of RA‐associated SNPs with the above outcomes are presented in Supplementary Table 2 (https://onlinelibrary.wiley.com/doi/10.1002/art.42239).

**Table 1:** Included studies and consortia*

Data source/outcome	Outcome	Ancestry	No. of patients	No. of controls	Adjustments in the GWAS
CARDIoGRAMplusC4D and UKBB					
CAD		Mixed	122,733	424,528	Not reported
MEGASTROKE consortium					
All stroke		European	40,585	406,111	Age and sex
Any ischemic stroke		European	34,217	406,111	Age and sex
Large artery stroke		European	3,373	406,111	Age and sex
Small vessel stroke		European	5,386	406,111	Age and sex
Cardioembolic stroke		European	7,193	406,111	Age and sex
ISGC Intracerebral hemorrhage		European	3,223	3,725	Age, sex, and principal components
GWAS by Bakker et al Subarachnoid hemorrhage		European	5,140	71,952	Not reported
UKBB					
All stroke		European	12,036	355,525	Age, sex, and 10 genetic principal components
Any ischemic stroke		European	6,566	360,995	Age, sex, and 10 genetic principal components
Intracerebral hemorrhage		European	1,504	366,057	Age, sex, and 10 genetic principal components
Subarachnoid hemorrhage		European	1,292	366,269	Age, sex, and 10 genetic principal components
FinnGen consortium					
CAD		European	30,952	187,840	Age, sex, first 10 genetic principal components, and genotyping batch
All stroke		European	18,661	166,201	Age, sex, first 10 genetic principal components, and genotyping batch
Any ischemic stroke		European	10,551	202,223	Age, sex, first 10 genetic principal components, and genotyping batch
Intracerebral hemorrhage		European	1,687	201,146	Age, sex, first 10 genetic principal components, and genotyping batch
Subarachnoid hemorrhage		European	1,338	201,230	Age, sex, first 10 genetic principal components, and genotyping batch

* GWAS = genome‐wide association study; CARDIoGRAMplusC4D = Coronary Artery Disease Genome‐wide Replication and Meta‐analysis plus The Coronary Artery Disease Genetics consortium; UKBB = UK Biobank; CAD = coronary artery disease; ISGC = International Stroke Genetic Consortium.

### Data sources for cardiovascular risk factors, inflammatory biomarkers, and inflammatory bowel disease (IBD)

We obtained summary‐level data for cardiovascular risk factors (including body mass index [22], blood pressure [23], fasting glucose and insulin [24], high‐density and low‐density lipoprotein cholesterol and triglyceride [25], smoking initiation [26], and moderate‐to‐vigorous physical activity [the Neale Lab data, http://www.nealelab.is/uk-biobank] and inflammatory biomarkers such as interleukin‐6 [IL‐6] [27], tumor necrosis factor [TNF] [27], and C‐reactive protein [CRP] [28]) from international consortia and the UK Biobank (the Neale Lab data). Summary‐level data on IBD were obtained from a genome‐wide association meta‐analysis study including 59,957 individuals of European descent (29). Detailed information on the studies used are shown in Supplementary Table 3 (https://onlinelibrary.wiley.com/doi/10.1002/art.42239).

### Genetic correlation analysis

Genome‐wide pairwise correlations between RA and studied CVD outcomes based on consortia data were estimated using linkage disequilibrium score regression (LDSC) that leverages genome‐wide association analysis summary‐level data and linkage disequilibrium to estimate genetic correlation (30). This method estimates universal genetic correlation by measuring correlation of effect size between SNP exposure and SNP outcome associations across all genetic variants in the genome. A genetic correlation (*rg*) of >0.7 was deemed a strong correlation. *P* values less than 0.006 (0.05 for 8 outcomes) was treated as significant in LDSC analysis.

### Statistical analysis

We aligned the SNPs based on allele letter and allele frequency. SNPs that were missing in the outcome data sets were replaced by proxy SNPs, which were searched in https://ldlink.nci.nih.gov/ by implementing a setting of *r* ^2^ > 0.8 and using European populations as reference groups. Missing SNPs without proxies were excluded from the analysis. We searched phenotypes associated with RA‐associated SNPs at the genome‐wide significance level in PhenoScanner V2, a database of human genotype–phenotype associations (31).

The inverse variance–weighted method under the multiplicative random effects model was used as the main method to calculate the associations of genetic liability to RA with cardiovascular outcomes, cardiovascular risk factors, and inflammatory biomarkers. This method can provide the most precise estimate; however, it is sensitive to horizontal pleiotropy and outliers. Several sensitivity analyses, including the weighted median (32), MR‐Egger (33), MR‐PRESSO (34), and contamination mixture (35) methods, were used to examine the consistency of results and detect and correct for horizontal pleiotropy. The weighted median analysis can provide consistent causal estimates, given that more than half of weight derives from valid SNPs (32). The MR‐Egger regression can detect the horizontal pleiotropy by its intercept test and provide estimates after correcting for pleiotropic effects; however, the analysis is less powerful for most scenarios (33). In a comparative study, power to detect causal effect is usually greater for the inverse variance–weighted method compared to the MR‐Egger method in scenarios of different status of pleiotropy and satisfaction of the Instrument Strength Independent of Direct Effect assumption (33). The MR‐PRESSO method can also correct for horizontal pleiotropy by identifying and removing outlying SNPs (34). The contamination mixture method is good at analysis based on multiple genetic instruments and can generate causal estimates even when instruments contain invalid SNPs (35).

In addition, we used scatter plots to visualize the heterogeneity in estimates of the SNPs used and to determine whether the association was driven by certain SNPs. Estimates from different data sets, but for the same CVD, were combined using the fixed‐effects meta‐analysis method in which study‐specific estimates were weighted based on the amount of information captured by that study (i.e., more weight was given to a large study with many patients than a small study with few patients). Given that the HLA gene regions are shared by RA and other autoimmune disorders (36), we performed a sensitivity analysis after removal of SNPs in these gene regions (including HLA–A, HLA–B, HLA–C, HLA–DPA1, HLA–DPB1, HLA–DQA1, HLA–DQB1, HLA–DRA, HLA–DRB1, and HLA–DRB3). We used the multivariable MR analysis to estimate the mediation effects of RA‐associated factors in the associations between RA and cardiovascular outcomes. The multivariable MR analysis was based on the same set of genetic instruments (SNPs for RA), and the model was based on summary‐level beta coefficients and the corresponding standard error for RA, the outcome, and the mediator. In addition, we conducted a multivariable MR analysis to adjust for genetic liability to IBD (a common autoimmune disease) to minimize its influence. Likewise, this analysis used the same genetic variants as the main analysis, and MR estimates were obtained from a multivariable inverse variance–weighted analysis on the association between genetic liability to RA with a CVD outcome with adjustment for genetic liability to IBD.

Cochran's Q statistic and *P* value for MR‐Egger intercept were used to assess the heterogeneity and horizontal pleiotropy, respectively. The Bonferroni correction was used to account for multiple testing in examining the association between RA and CVDs. Associations with a *P* value less than 0.006 (0.05 for 8 outcomes) were deemed significant in order to correct for multiple testing. All tests were 2‐sided and were conducted using the TwoSampleMR and MendelianRandomization package (37, 38).

## RESULTS

Results of the search in PhenoScanner V2 are presented in Supplementary Table 4 (https://onlinelibrary.wiley.com/doi/10.1002/art.42239). Several RA‐associated SNPs were found to be associated with other autoimmune diseases, including IBD, systemic lupus erythematosus, and type 1 diabetes, at the genome‐wide significance levels. A few other traits, such as immune cells, were identified to be associated with the SNPs that were used. There were few strong genetic correlations between RA and the studied cardiovascular outcomes (Supplementary Table 5, https://onlinelibrary.wiley.com/doi/10.1002/art.42239). RA showed a weak significant association with overall stroke (*rg* = 0.20; *P* = 0.003).

**Figure 1:**
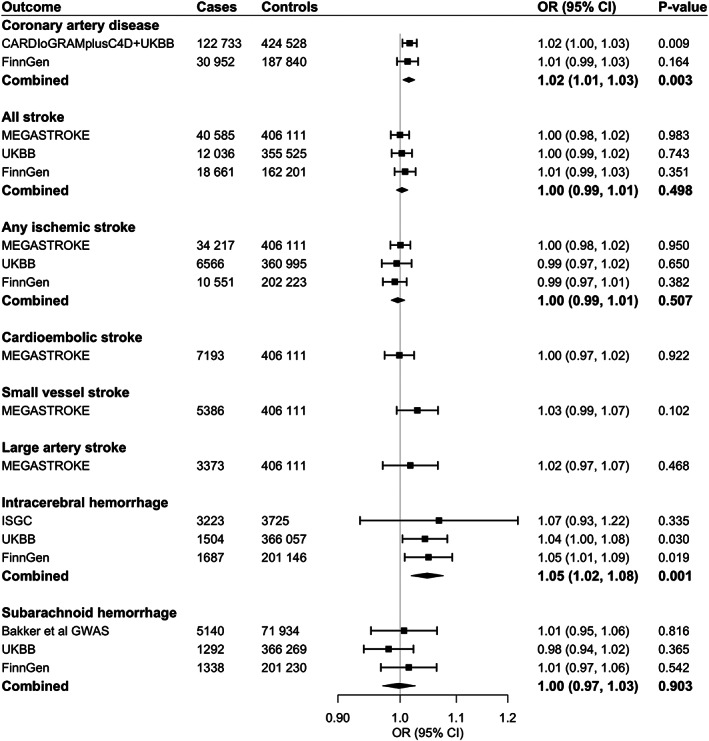
Associations of genetic liability to rheumatoid arthritis with coronary artery disease and stroke. OR = odds ratio; 95% CI = 95% confidence interval; CARDIoGRAMplusC4D = Coronary Artery Disease Genome‐wide Replication and Meta‐analysis plus The Coronary Artery Disease Genetics consortium; UKBB = UK Biobank; ISGC = International Stroke Genetic Consortium; GWAS = genome‐wide association study.

Genetic liability to RA was associated with an increased risk of CAD and ICH (Figure 1) consistently across sources. For a 1‐unit increase in log odds of RA, the combined odds ratios (ORs) were 1.02 (95% confidence interval [95% CI] 1.01, 1.03; *P* = 0.003) for CAD and 1.05 (95% CI 1.02, 1.08; *P* = 0.001) for ICH. The results were stable in all sensitivity analyses (Supplementary Table 6, https://onlinelibrary.wiley.com/doi/10.1002/art.42239). In a supplementary analysis in which estimates for the CVD outcomes were scaled per 1% increase in genetic liability to RA on the risk difference scale, the OR was 1.03 (95% CI 1.01, 1.05) for CAD and 1.06 (95% CI, 1.01, 1.11) for ICH (Supplementary Table 7, https://onlinelibrary.wiley.com/doi/10.1002/art.42239).

**Figure 2:**
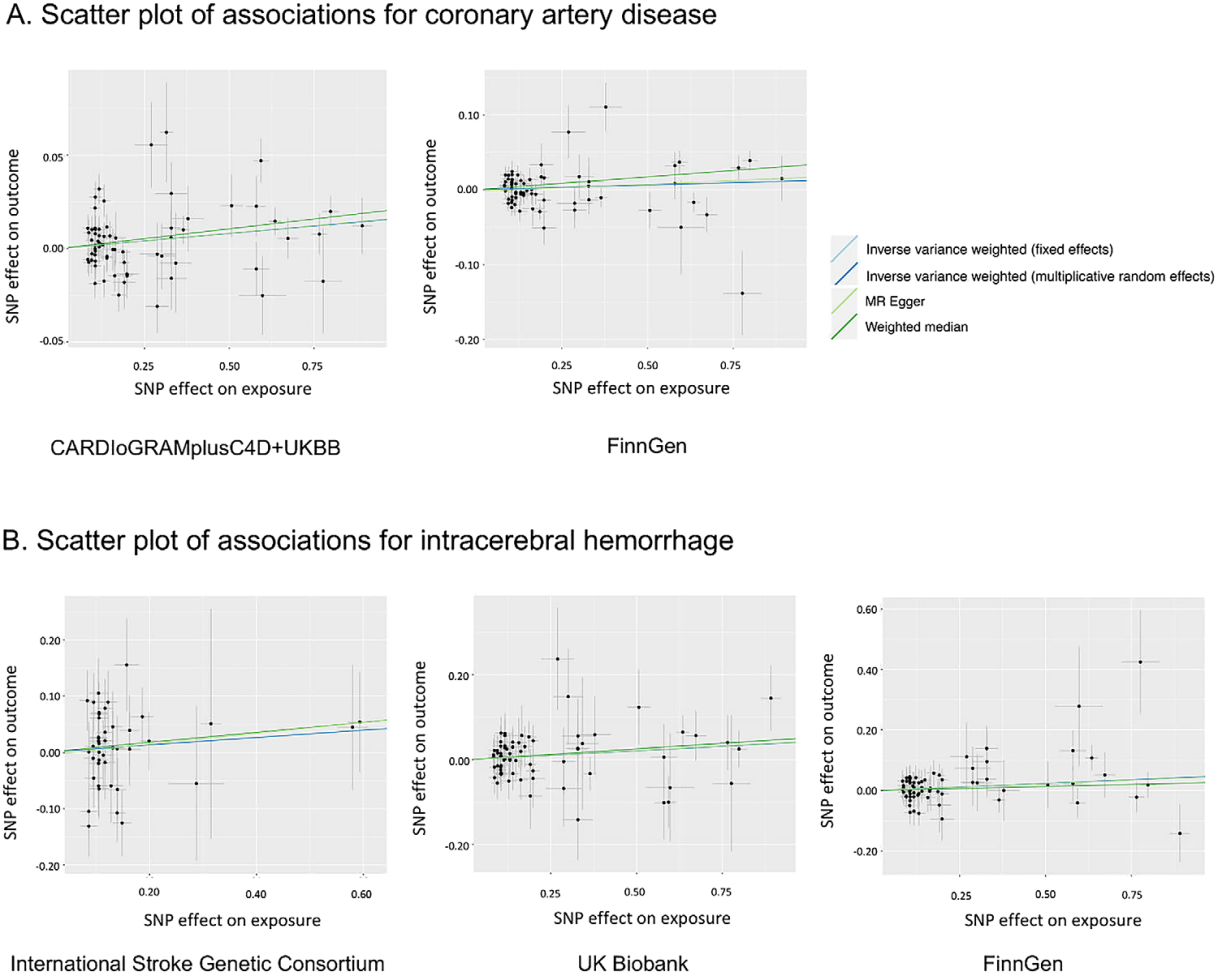
Scatter plots of associations with coronary artery disease and intracerebral hemorrhage. SNP = single‐nucleotide polymorphism; CARDIoGRAMplusC4D = Coronary Artery Disease Genome‐wide Replication and Meta‐analysis plus The Coronary Artery Disease Genetics consortium; MR = Mendelian randomization.

We detected moderate heterogeneity in the analyses for CAD and no horizontal pleiotropy (*P* for MR‐Egger intercept test > 0.4) (Supplementary Table 6). Even though a few outliers were detected in the MR‐PRESSO analyses for CAD, the associations remained consistent after removal of these outliers (Supplementary Table 6). As for associations with ICH in the 3 data sets, we observed no or modest heterogeneity, no indication of horizontal pleiotropy in the MR‐Egger intercept tests, and no outliers were detected by the MR‐PRESSO analyses (Supplementary Table 6). In scatter plots of associations with CAD and ICH, we did not observe any SNPs that drove the overall positive associations (Figure 2). Otherwise, there were no associations of genetic liability to RA with all stroke, any ischemic stroke and its subtypes, or subarachnoid hemorrhage (Figure 1 and Supplementary Table 6).

The observed associations with CAD and ICH remained stable in the sensitivity analysis after removal of SNPs in HLA gene regions (Supplementary Table 8, https://onlinelibrary.wiley.com/doi/10.1002/art.42239). The associations were also stable in the multivariable MR analysis with adjustment for genetic liability to IBD (Supplementary Table 9, https://onlinelibrary.wiley.com/doi/10.1002/art.42239).

**Figure 3:**
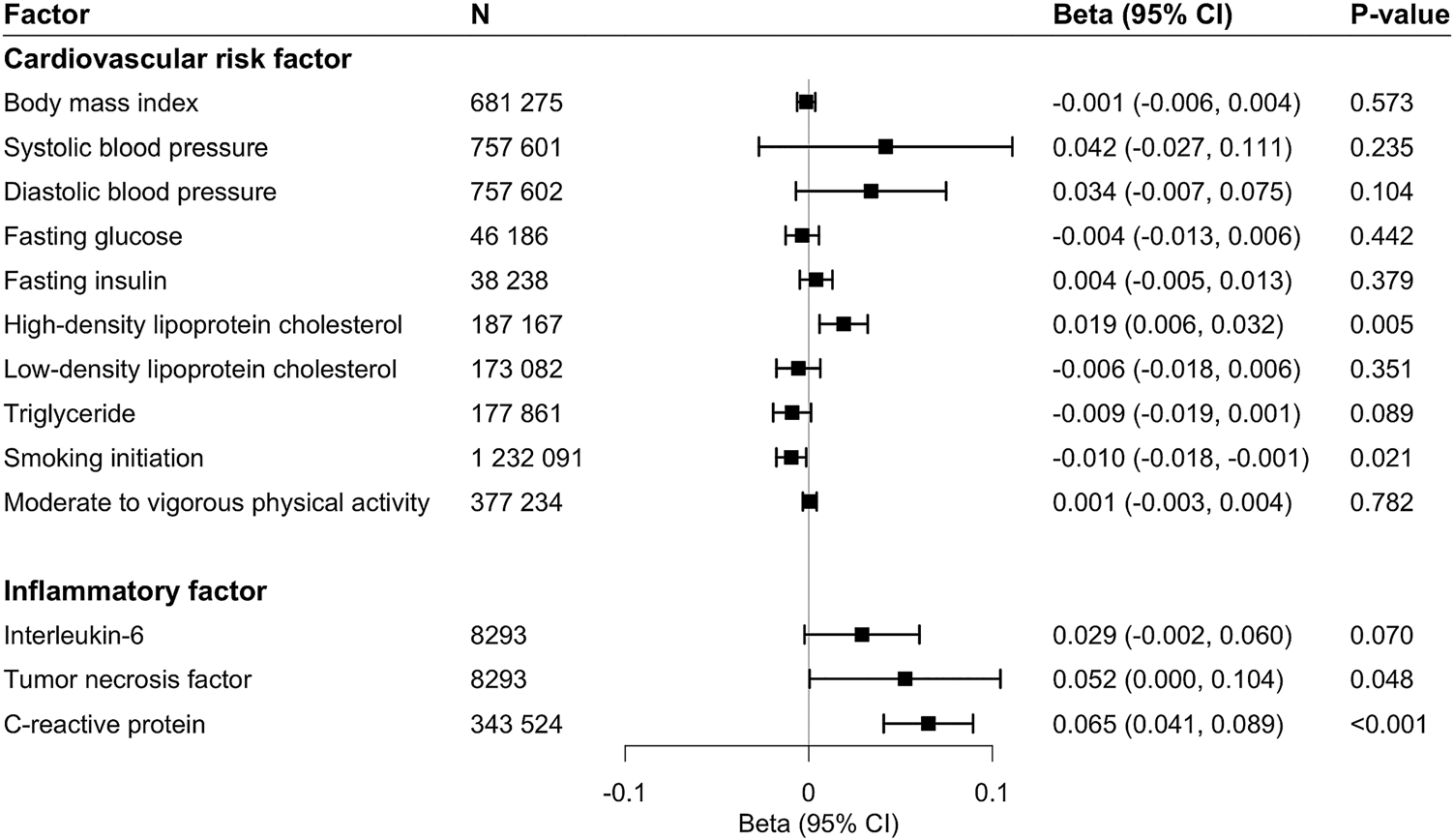
Associations of genetic liability to rheumatoid arthritis with cardiometabolic risk factors and inflammatory cytokines. 95% CI = 95% confidence interval.

With respect to cardiometabolic risk factors, genetic liability to RA was associated with reduced log odds ratio of smoking initiation and increased levels of high‐density lipoprotein cholesterol, TNF, and CRP (Figure 3). The associations remained directionally consistent in sensitivity analyses (Supplementary Table 10, https://onlinelibrary.wiley.com/doi/10.1002/art.42239). There were no associations of genetic liability to RA with the other cardiovascular risk factors and inflammatory biomarkers studied (Figure 3).

**Figure 4:**
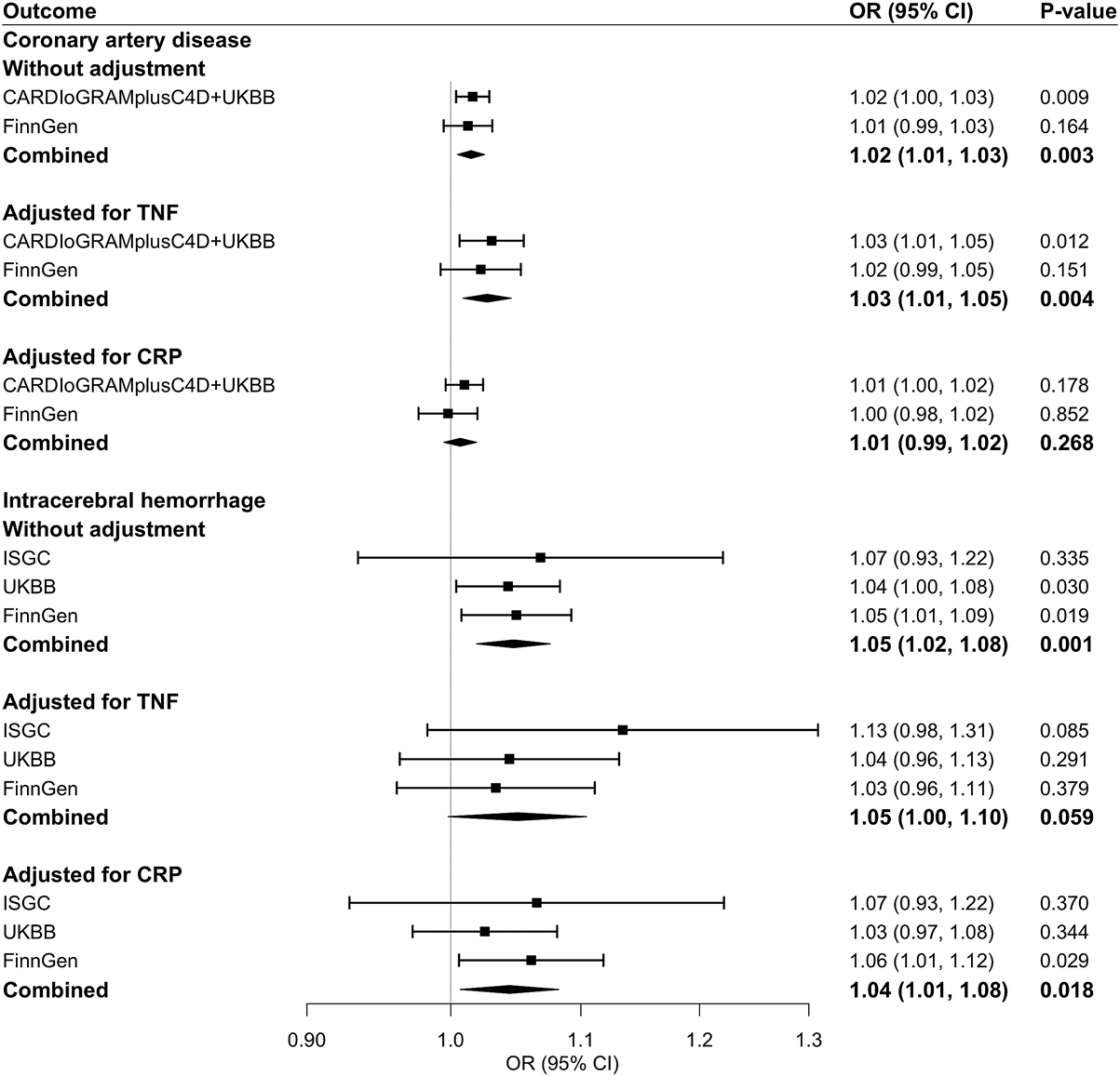
Associations of genetic liability to rheumatoid arthritis with coronary artery disease and intracerebral hemorrhage after adjustment for tumor necrosis factor (TNF) and C‐reactive protein (CRP). See Figure 1 for other definitions.

Multivariate MR analyses were conducted to adjust for genetically predicted levels of TNF and CRP levels. The association between RA and CAD attenuated in the analysis with adjustment for genetically predicted CRP levels but not in the analysis with adjustment for genetically predicted TNF. The association between RA and ICH changed only slightly in the multivariable MR analyses (Figure 4).

## DISCUSSION

We conducted a 2‐sample MR study to investigate the causal associations of RA with CAD and stroke using data from large consortia and genetic studies. Few strong genetic correlations were observed between RA and studied cardiovascular outcomes. We found that genetic liability to RA was associated with elevated risk of CAD and ICH but not ischemic stroke or subarachnoid hemorrhage. These associations were consistent across different data sources, after removal of SNPs in HLA gene regions, and in the multivariable MR analysis with adjustment for genetic liability to IBD. Genetic liability to RA was associated with elevated levels of TNF and CRP. The increased levels of CRP appeared to mediate the association with CAD. We thus provide important genetic evidence supporting the link between RA and some CVDs and underscore the role of inflammation in driving CAD specifically.

RA has widely been reported as an important risk factor for CAD and impaired vascular function (33). A higher prevalence, extent, and severity of coronary plaque measured by coronary calcification (6) is found in RA patients, and this is related to disease duration, being increased in established compared to early RA (31). Similarly, invasive angiographic studies have also demonstrated RA to be associated with an increased extent of coronary atherosclerosis, with a higher prevalence of multivessel CAD, even after adjustment for some traditional risk factors (9). Importantly, this accelerated coronary atherosclerosis also appears to confer a substantially elevated risk of cardiovascular events, with incident myocardial infarction and CAD‐related mortality increased by 68% and 59% in RA, respectively, according to large meta‐analyses (3, 4).

Our findings support a causal role for RA in driving CAD, although we report a more modest effect size of 2% in the main analysis. This likely relates to differences in outcome definition (myocardial infarction versus the softer end point of CAD), more healthy populations included, and the calculation of risk according to log odds of RA. Overall, though, the totality of evidence suggests CAD to be increased in RA, and our study strongly suggests a causal role in this. There are ~14 million people globally with RA (39). As lifetime risk of CAD for people in general is already high (40), even a small increase in odds of CVD raises the expected number of CAD events in this population by tens of thousands compared to that expected for a similar sized group without RA. This potentially impacts public health policy around targeting of education, screening, and treatment at RA patients. Second, it provides insight into potential mechanisms of CAD. Even though we identified that chronic inflammation might mediate the association between RA and CAD, future research may be able to pinpoint exactly which metabolic or inflammatory changes occur in people with RA that lead to this increase in CAD, potentially identifying treatment or screening options for the whole population.

Stroke risk, of both ischemic and hemorrhagic types, has also widely been reported in observational studies to be increased in RA patients (41). Our study did not detect an association between genetic liability to RA and risk of stroke overall or ischemic stroke. This discrepancy may be related to confounding in observational studies, or increased stroke risk may not be caused by RA per se but by certain features of RA patients. Consistent with this, incident adverse events, including serious infections and insufficient treatment of CVDs, have been found to be drivers of the increased risk of stroke in RA patients (42). These null MR findings might be caused by inadequate power. Our study did, however, suggest a causal role for RA in causing ICH and supports the 68% increased risk reported in meta‐analysis of observational studies (41). Our consistent findings across 3 data sets highly suggest the validity of this association, and the underlying mechanisms warrant further investigation.

Several mechanisms have been proposed to explain the increased risk of CVD in RA patients. First, it has been suggested that RA may influence the development of traditional cardiovascular risk factors. We did not find genetic liability to RA to be associated with the majority of cardiometabolic risk factors, and only high‐density lipoprotein cholesterol, a protective factor, was significantly increased. We therefore provide some mechanistic evidence against the role of traditional risk factors, although it is possible that RA may indirectly influence traditional risk factors, such as due to side effects of antirheumatic or antiinflammatory medications. In addition, our null MR findings for the associations of genetic liability to RA with cardiometabolic risk factors could not completely rule out the effects of shared nongenetic factors on these associations. Furthermore, although far from significant, relatively large effect sizes were found for blood pressure in our study. In a recent published MR analysis including 461,880 hypertension patients and 337,653 controls, RA was associated with a high risk of hypertension (43), which is an important risk factor for CAD and the main cause of hemorrhagic stroke. However, a key hypothesis is that elevated systemic inflammation and remarkably overlapping inflammatory processes between the 2 conditions lead to progression of CVD (11, 44). In agreement with this, circulating levels of inflammatory markers such as CRP, erythrocyte sedimentation rate, and IL‐6 in RA patients are associated with a risk of cardiovascular events (11, 45) and with radiologic measures of coronary atherosclerosis (46).

Our study supports the notion that chronic inflammation drives CAD risk in RA, as CRP mediates the association between RA and CAD risk. However, our multivariable MR analysis did not suggest an important role of TNF for CAD or for RA‐associated inflammation on ICH. Other RA‐related abnormalities that may predispose one to CAD or ICH may include endothelial dysfunction, oxidative stress, lipid alterations, and posttranslational modifications of peptides (45). Further investigation is required into the mechanisms underlying the association between RA and ICH.

Elevated CVD in RA has long been recognized, and in recent years this has been incorporated into European clinical guidelines written for use by both rheumatology (European Alliance of Associations for Rheumatology) (47) and cardiology (European Society of Cardiology) (48) clinicians. In particular, the importance of regular cardiovascular risk assessment every 5 years is emphasized, as is the use of a 1.5‐fold multiplication factor to account for RA in risk scores based solely on traditional risk factors. However, evidence exists that cardiovascular risk factors remain undiagnosed in RA patients, and, even when detected, they may be undermanaged compared to patients with other risk factors like diabetes (49). Our study provides the first MR evidence supporting a causal role of RA in driving heightened cardiovascular risk. This not only emphasizes the importance of monitoring this high‐risk population, but also supports the notion in clinical guidelines that combating rheumatic disease activity is also integral to reducing cardiovascular risk. Current strategies to do this remain controversial, as some therapies have been associated with adverse cardiovascular effects (47, 50).

We provide the causal genetic evidence that inflammation drives CAD risk in RA and implicate this as an effective therapeutic target. TNF inhibitors are commonly used in clinical practice, and although some evidence exists for reduced cardiovascular risk in patients on such treatments (51), our results do not support this. CRP is a broad inflammatory marker raised by many pathways, including the IL‐1/IL‐6 axis. IL‐1 inhibition reduced cardiovascular events in the Canakinumab Antiinflammatory Thrombosis Outcome Study trial, and IL‐6 inhibition has been found to have beneficial effects on markers of atherosclerosis such as carotid intima‐media thickness (47) and to reduce cardiovascular events in RA (52). Although antiinflammatory treatments may prove useful in cardiovascular prevention in RA, studies to date have had inadequate follow‐up times and have been confounded by therapies being allocated to those with the most severe disease. Well‐designed clinical trials studying the impact of antiinflammatory therapies on cardiovascular risk in RA are required.

Given that autoimmune diseases have some overlapping genetic architecture, whether the observed associations between RA and CAD and between RA and ICH in our MR analysis were exclusive to RA remained undetermined, even though we employed several approaches to examine this. First, the results from the search of phenotypes associated with RA‐associated SNPs in PhenoScanner V2 showed no clear pattern that these used RA‐associated SNPs could systematically mimic the effects of other immune‐mediated disorders, although several RA‐associated SNPs were associated with several other immune‐mediated diseases at the genome‐wide significance level. Second, the observed associations with CAD and ICH remained stable in the sensitivity analysis after removal of SNPs in the HLA gene regions shared by autoimmune disorders (36), which indicated that the effects of most shared genes among autoimmune diseases did not drive the associations. Third, the associations remained in the multivariable MR analysis with adjustment for IBD. However, we could not perform this analysis to adjust for genetic liability to other common autoimmune diseases due to lack of data or too many missing SNPs in corresponding analysis. Even though our exploration implies the observed associations with CAD and ICH are likely to be specific to RA, further studies are needed to confirm our hypothesis.

The present study has several strengths, including MR design, the use of multiple genetic instruments, the use of different outcome data sources, the use of the multivariable MR analysis to explore possible mechanisms, and the population confinement to individuals of European descent (reducing population structure bias). In addition, lack of strong genetic correlations between RA and studied outcomes suggest that the observed associations with CAD and ICH may not be driven by shared genetic risk.

Several limitations should be considered when interpreting our findings. We observed moderate heterogeneity in the analyses for CAD in the CARDIoGRAMplusC4D (Coronary Artery Disease Genome‐wide Replication and Meta‐analysis plus The Coronary Artery Disease Genetics) consortium, UK Biobank, and FinnGen data sets. However, the corresponding MR‐Egger regression analysis did not detect any indication of horizontal pleiotropy, which suggests possible balanced horizontal pleiotropy that is unlikely to bias the MR estimate (53). In addition, the associations with CAD in 2 data sets were consistent across different sensitivity analyses with different assumptions. Even though there were a few outliers detected by MR‐PRESSO analyses, the associations remained after removal of these outliers. We did not take anti‐RA treatments into consideration in the current analysis. Nonetheless, whether corresponding treatments, such as anti‐TNF drugs and nonsteroidal antiinflammatory drugs, are associated with cardiovascular risk is unclear (44, 54). In addition, these treatments should not bias our causal estimation, as their use follows the diagnosis of RA and would therefore be classified as vertical pleiotropy (53).

The population confinement to European populations might limit the generalizability of our findings to other populations. In addition, whether the null findings for stroke and its subtypes (except for ICH) could be robustly held are uncertain, as the lack of significant associations might be caused by inadequate power despite the large sample size, at least for ischemic stroke. A power calculation for the current analysis was not possible due to the lack of information on phenotypic variance in RA explained by the SNPs used in the analysis, as this information cannot be calculated for a binary phenotype. Thus, future studies are needed to confirm these null findings. Whether the observed associations could be applied to subgroups defined by sex and status of anti–citrullinated protein autoantibodies could not be assessed due to lack of data.

In conclusion, this MR study found positive associations of genetic liability to RA with CAD and ICH, and the association with CAD appeared to be mediated by high levels of CRP. These findings highlight the importance of active monitoring and prevention of cardiovascular risk to combat CAD and ICH in RA patients. We further suggest that dampening inflammation might be a preventive strategy for CAD in RA patients, and well‐designed clinical trials are required to assess this.

## AUTHOR CONTRIBUTIONS

All authors were involved in drafting the article or revising it critically for important intellectual content, and all authors approved the final version to be published. Dr. Yuan had full access to all of the data in the study and takes responsibility for the integrity of the data and the accuracy of the data analysis.

### Study conception and design

Yuan, Carter, Larsson.

### Acquisition of data

Yuan, Carter, Mason, Burgess, Larsson.

### Analysis and interpretation of data

Yuan, Carter, Mason, Yang, Burgess, Larsson.

## Supporting information


Disclosureform

**Table S1** Genetic instruments for rheumatoid arthritis
**Table S2**. Associations of RA‐associated SNPs with studied outcomes
**Table S3**. Data sources for cardiovascular risk factors and inflammatory biomarkers
**Table S4**. Phenotypes assiciated with genetic instruments at the genome‐wide significance level
**Table S5**. Genetic correlations between RA and studied outcomes
**Table S6**. Associations of genetic liability to rheumatoid arthritis with coronary artery disease and stroke in sensitivity analyses
**Table S7**. Odds ratios of coronary artery disease and stroke per 1% increase in genetic liability to RA in the inverse variance weighted method
**Table S8**. Associations of genetic liability to rheumatoid arthritis with coronary artery disease and intracerebral hemorrhage after removal of SNPs in HLA gene regions
**Table S9**. Associations of genetic liability to rheumatoid arthritis with CAD and intracerebral hemorrhage in the multivariable MR analysis with adjustment for genetic liability to inflammatory bowel disease
**Table S10**. Associations of genetic liability to rheumatoid arthritis with cardiovascular risk factors and inflammatory biomarkers in sensitivity analyses
