# Supplementary material for: Genetic Liability to Rheumatoid Arthritis in Relation to Coronary Artery Disease and Stroke Risk
Source: Arthritis Rheumatol. 2022 Aug 17;74(10):1638–47. doi: 10.1002/art.42239 (PMC9804931; doi:10.1002/art.42239)

Supplementary Tables for

**Genetic liability to rheumatoid arthritis in relation to coronary artery disease and stroke risk**

Shuai Yuan, Paul Carter, Amy M. Mason, Fangkun Yang, Stephen Burgess, Susanna C. Larsson

| ST 1. Genetic instruments for rheumatoid arthritis |
| --- |
| ST 2. Associations of RA-associated SNPs with studied outcomes |
| ST 3. Data sources for cardiovascular risk factors and inflammatory biomarkers |
| ST 4. Phenotypes assiciated with genetic instruments at the genome-wide significance level |
| ST 5. Genetic correlations between RA and studied outcomes |
| ST 6. Associations of genetic liability to rheumatoid arthritis with coronary artery disease and stroke in sensitivity analyses |
| ST 7. Odds ratios of coronary artery disease and stroke per 1% increase in genetic liability to RA in the inverse variance weighted method |
| ST 8. Associations of genetic liability to rheumatoid arthritis with coronary artery disease and intracerebral hemorrhage after removal of SNPs in HLA gene regions |
| ST 9. Associations of genetic liability to rheumatoid arthritis with CAD and intracerebral hemorrhage in the multivariable MR analysis with adjustment for genetic liability to inflammatory bowel disease |
| ST 10. Associations of genetic liability to rheumatoid arthritis with cardiovascular risk factors and inflammatory biomarkers in sensitivity analyses |

All tables can be obtained in OSF data respiratory: <https://osf.io/xnf3h/?view_only=9d4de76b50b94897be6006e361e8f92f>

Please view the file by click the xlsx file.


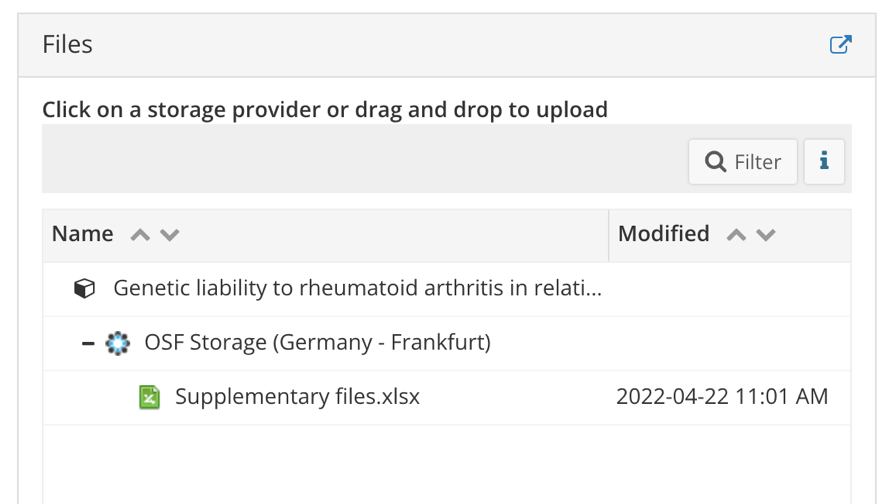

Supplement: Supplementary file 2 — Table S1 Genetic instruments for rheumatoid arthritis Table S2. Associations of RA‐associated SNPs with studied outcomes Table S3. Data sources for cardiovascular risk factors and inflammatory biomarkers Table S4. Phenotypes assiciated with genetic instruments at the genome‐wide significance level Table S5. Genetic correlations between RA and studied outcomes Table S6. Associations of genetic liability to rheumatoid arthritis with coronary artery disease and stroke in sensitivity analyses Table S7. Odds ratios of coronary artery disease and stroke per 1% increase in genetic liability to RA in the inverse variance weighted method Table S8. Associations of genetic liability to rheumatoid arthritis with coronary artery disease and intracerebral hemorrhage after removal of SNPs in HLA gene regions Table S9. Associations of genetic liability to rheumatoid arthritis with CAD and intracerebral hemorrhage in the multivariable MR analysis with adjustment for genetic liability to inflammatory bowel disease Table S10. Associations of genetic liability to rheumatoid arthritis with cardiovascular risk factors and inflammatory biomarkers in sensitivity analyses [file ART-74-1638-s001.zip › art42239-sup-0002-TableS1.docx]
